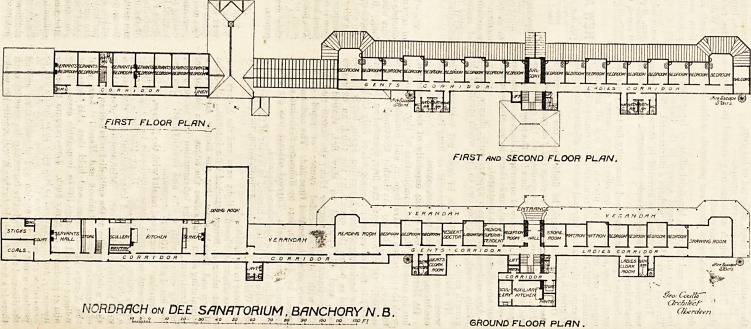# Nordrach-On-Dee Sanatorium, Banchory, near Aberdeen

**Published:** 1901-06-01

**Authors:** 


### The Institutional Workshop.

### NORDRACH-ON-DEE SANATORIUM, BANCHORY, NEAR ABERDEEN.

This Sanatorium has been specially designed and built with the object of carrying out in North Britain the open-air treatment of consumption; and as the cost of the building and its appurtenances reached the high sum of £650 per bed, it is evident that no expense has been spared in bringing it thoroughly up to date and making it worthy of the important object in view.

The Sanatorium is built in linear form. By this arrangement it is a simple matter to separate the administrative department from the hospital proper, and by it it is also possible to ensure the maximum of sunshine. A wide verandah surrounds, on three aspects, the hospital section of the building, and this section is divided into two parts, one for ladies and the other for men. The building is three stories in height.

All the bedrooms are so arranged that they will admit the greatest amount of sunshine and of air and light. The windows occupy more than two-thirds of the outer-wall space, and they are so constructed that they may be kept open during all weathers. The floors are made of polished wood whereby absorption is reduced to a minimum, and the corners of the rooms are rounded off, thus doing away with very common dust-traps. Every bedroom is fitted up with its own douche bath and fixed lavatory basins with cold and hot supplies of water. The electric light is in use throughout.

In all cases the sanitary blocks are spurs of the main building, and have cross-ventilated passages. The dining-hall is placed between the administrative part and the hospital. It is a fine room, measuring 56 feet by 24 feet.

The Sanatorium is built in the middle of a pine forest on the southern slope of the mountain, commanding beautiful and extensive views. On the north rises the Hill of Fare; on the south is Goch Hill, and in the far west lies the Grampian range. During nine months of the year, or thereabouts, south-west winds prevail along Deeside, and it is this range of mountains that intercepts much of the moisture with which these winds are usually laden, ensuring a fairly dry climate. The subsoil is red gravel. For any human habitation this possesses many advantages, not the least of which are that land drainage is easily carried out and that the surface is soon dry, even after a heavy rainfall.

As already said, the air in this district is dry the temperature comparatively high (on some occasions its winter temperature has been known to be higher than that of the South of England), and it is very rich in ozone. The percentage of sunshine in Deeside is known to be above the average, and to all these advantages may be added the odour of the pine woods. It is possible these odours may possess some medicinal properties; it is certain they are pleasant to our sense of smell. The rainfall is between twenty-six and thirty inches a year. There is less snow than in the English Midlands, and there is an almost total absence of fog. When the weather is cold it is a dry cold, hence much more easily borne, a fact of great importance to the consumptive patient who has to spend the whole day out of doors. Banchory is about eighteen miles from Aberdeen, and as there is no large centre of population nearer than that city, there can be no danger of air-contamination from such sources.

The treatment pursued at Banchory does not sensibly differ from that followed at the best English and foreign hospitals for consumption, and it is carried out by Drs. Lawson and Bardswell. There is a fully-equipped laboratory placed in charge of Mr. J. E. Chapman. In many cases it is certain that the bacteriological investigations will be of use in helping the physicians to determine the finer points of diagnosis, and hence conduce to a more correct treatment than might be possible in the absence of such microscopic and analytical work.

Competent nurses, a masseuse and a masseur, reside on the premises.

The terms (almost inclusive) are five guineas a week.

Aberdeen may be reached in about twelve hours from London.

## Figures and Tables

**Figure f1:**